# MiR-200c is a cMyc-activated miRNA that promotes nasopharyngeal carcinoma by downregulating PTEN

**DOI:** 10.18632/oncotarget.14123

**Published:** 2016-12-23

**Authors:** Pan Chen, Xiaofang Guo, Liming Zhang, Wenling Zhang, Qingyu Zhou, Zhi Tian, Ying Zheng, Qianjin Liao, Heran Wang, Guiyuan Li, Jin Huang, Xiayu Li

**Affiliations:** ^1^ Hunan Cancer Hospital and The Affiliated Cancer Hospital of Xiangya School of Medicine, Central South University, Changsha, Hunan 410013, China; ^2^ Key Laboratory of Carcinogenesis of Ministry of Health, Cancer Research Institute, Central South University, Xiangya Road, Changsha, Hunan 410078, China; ^3^ Department of Pharmaceutical Sciences, College of Pharmacy, University of South Florida, Tampa, FL33612, USA; ^4^ The Department of Laboratory Medicine, Huaihua Medical College, Huaihua, Hunan 418000, China; ^5^ Department of Oncology, Xiangya Hospital, Central South University, Changsha, Hunan 410078, China; ^6^ Hunan Key Laboratory of Nonresolving Inflammation and Cancer, Disease Genome Research Center, The Third Xiangya Hospital, Central South University, Changsha, Hunan 410013, China

**Keywords:** miRNA-200c, c-Myc, PTEN, nasopharyngeal carcinoma

## Abstract

The c-Myc transcription factor regulates a complex transcriptional program that leads to cellular transformation by targeting a large number of protein-encoding genes and non-coding RNAs. In this study, we show that a microRNA, miR-200c, is a novel c-Myc target that promotes cellular transformation and metastasis in nasopharyngeal carcinoma. MiR-200c achieves this oncogenic effect, at least in part, by targeting and inhibiting the tumor suppressor gene *PTEN* (phosphatase and tensin homolog), which is a key inhibitor of the AKT kinase signaling that promotes tumorigenesis in nasopharyngeal carcinoma. Our study thus identifies cMyc-miR-200c-PTEN-AKT as a functional module that promotes cellular transformation in nasopharyngeal carcinoma.

## INTRODUCTION

The c-Myc oncogene encodes an evolutionarily conserved basic helix-loop-helix leucine zipper transcription factor that is commonly dysregulated in cancer, resulting in pleiotropic effects on cancer cell growth, proliferation, survival, angiogenesis, and metastasis [1, 2]. Recent studies have demonstrated that c-Myc exerts its oncogenic role by its ability to dramatically reprogramme microRNA (miRNA) expression that simultaneously modulate complex genetic networks including various oncogenes and tumor suppressor genes by inhibiting translation of their mRNAs [3–7]. Although many c-Myc targeted miRNAs have been implicated in cancer, newer miRNAs that have key roles in mediating tumorigenes is need to be identified and studied [8–11].

MiR-200c is a member of the miR-200 family which consists of miR-200a, miR-200b, and miR-200c, miR-141 and miR-429. In humans, miR-200a, miR-200b and miR-429 are located on chromosome 1 whereas miR-200c and miR-141 are located on chromosome 12 [12]. Members of the miR-200 family are highly enriched in epithelial tissues [13]. A recent study showed that miR-200c inhibited invasion and migration in human colon cancer cells (SW480/620) by targeting ZEB1 suggesting that it could be a metastasis suppressing miRNA which is opposite to our findings [14]. However, many studies have demonstrated that miR-200c is positively associated with malignancy of human cancers. Croce and colleagues found that the miR-200 family (miR-200a, miR-200b, miR-200c and miR-141) was upregulated in human ovarian cancers, especially in serous and Endometrioid histotypes [15]. A subsequent study confirmed this finding in serous ovarian cancers [16]. Moreover, overexpression of miR-200c in non-metastatic 4TO7 cells resulted in epithelial-mesenchymal transition (EMT) and colonisation of the liver and lung [17, 18].

In this study, we demonstrated that miR-200c, is a c-Myc regulated miRNA that influences oncogenic transformation by inhibiting the tumor suppressor gene *PTEN* resulting in subsequent activation of serine/threonine kinase, AKT.

## RESULTS

### c-Myc inhibition decreases miRNA-200c levels

**Figure 1:**
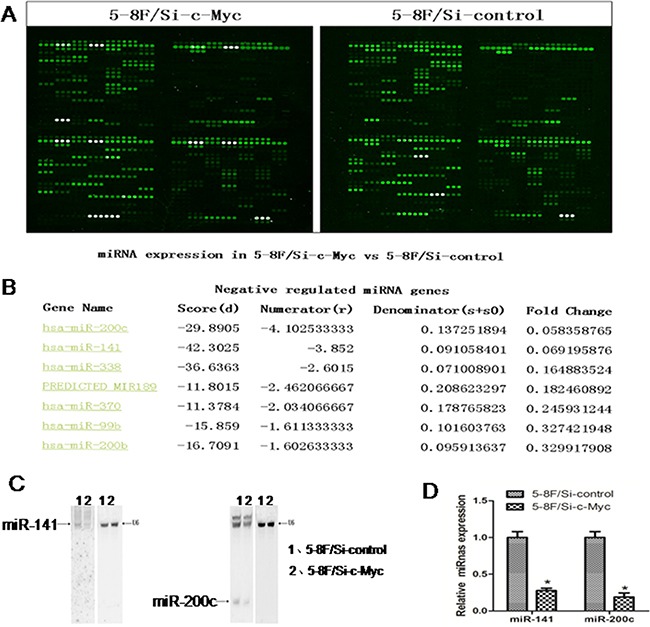
MicroRNA profile changes due to c-Myc inhibition in 5-8F NPC cells **A**. The miRNA microarray results for 5-8F/Si-c-Myc and 5-8F/Si-control cells. **B**. The fluorescence value for miR-200c from the miRNA microarray results. **C**. Northern blot analysis showing relative expression levels of miR-200c normalized to U6 (control) in the samples used for the microarray. **D**. Real-time PCR analysis showing relative expression levels of miR-200c normalized to U6 (control) in the samples used for the microarray chip.

To study the role of c-Myc in nasopharyngeal carcinoma (NPC), we used a stable NPC 5-8F cell line in which endogenous c-Myc was downregulated by siRNA [19]. We used a miRNA microarray to analyse the miRNA profile changes between the 5-8F/Si-c-Mycand the 5-8F/Si-control cell lines (Figure 1A). This miRNA microarray measures the expression of 434 human, 196 rat and 261 mouse miRNAs (Figure 1A). Our study found that upon c-Myc downregulation, twelve human miRNAs were upregulated and seven miRNAs were downregulated (with a fold change >3) in the 5-8F/Si-c-Myc cell line. The most dramatically downregulated miRNA was miR-200c that showed a 10 fold change. Since we had previously demonstrated the role of miR-216b in the nasopharyngeal carcinoma-related gene network [24], we wanted to explore the role of miR-200c in NPC. To exclude false-positives from the miRNA microarray analysis, we confirmed the downregulation of miR-200c in 5-8F/Si-c-Myc cells by Northern blot and RT-PCR analyses (Figures 1B-1D).

**Figure 2:**
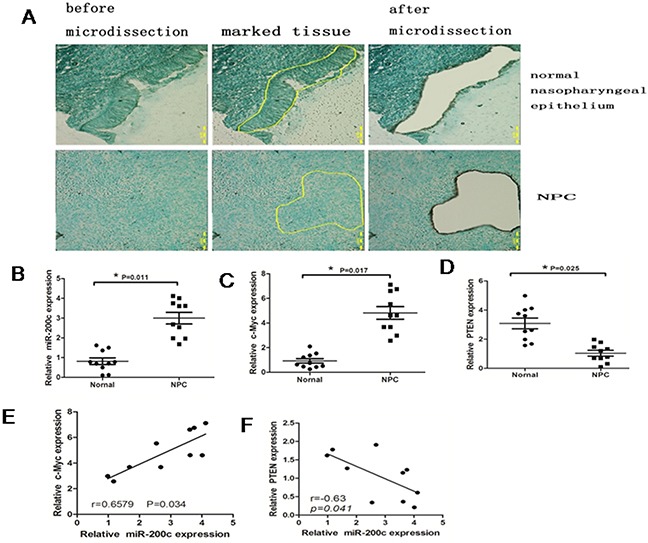
MiR-200c is upregulated in NPC specimens **A**. Top panel shows representative photographs (×100) of normal nasopharyngeal epithelium microdissection and the bottom panel shows NPC epithelium. The samples were stained with methyl green. **B**. Real-time PCR data showing relative expression levels of miR-200c in10 normal tissues and 10 NPC tissues. Data were normalized to U6 mRNA (control) and statistically analysed by unpaired Student's t-test (P =0.0021). **C**. Real-time PCR analysis showing relative expression levels of c-Mycin10 normal tissues and 10 NPC tissues. Data were normalised to that of GAPDH (control) and analysed by unpaired Student's t-test (P =0.0064). **D**. Real-time PCR analysis showing the relative expression levels of PTEN with 10 normal tissues and 10 NPC tissues. Data were normalised to that of GAPDH (control) and statistically analysed by unpaired Student's test (P=0.0075). **E**. The relationship between c-Myc and miR-200c. **F**. The relationship between miR-200c and PTEN.

Since c-Myc functions as an oncogene in NPC, we postulated that the role of miR-200c in 5-8F cells was to control its downstream targets. Therefore, we tested miR-200c expression in 10 NPC and 10 normal nasopharyngeal epithelium specimens by RT-PCR. The tissue samples were obtained by laser-capture microdissection and the lymphocytes and interstitial cells were removed prior to RNA extraction (Figure 2A). The results showed that the average expression levels of miR-200c and c-Myc were significantly higher and the average expression levels of PTEN were significantly lower in the NPC specimens than in the normal nasopharyngeal epithelium tissues (P<0.05; Figure 2B-2D). Moreover, the expression level of miR-200c was found to be directly correlated with the expression level of c-Myc (P < 0.05; Figure 2E), but inversely correlated with PTEN (P < 0.05; Figure 2F).

### MiR-200c increases cell cycle and promotes cellular migration and invasiveness

**Figure 3:**
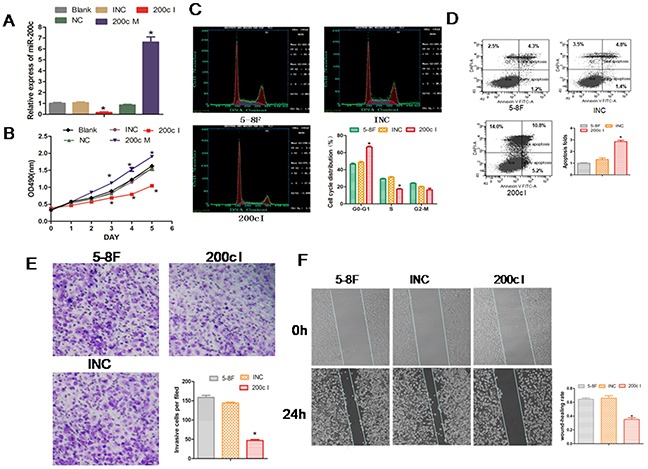
Inhibition of miR-200c expression reduces growth, cell cycle, invasiveness of 5-8F NPC cells and promotes cell apoptosis **A**. The 5-8F NPC cells were transfected with 200cM (mimic), 200cI (inhibitor) or their corresponding control miRNAs (NC or INC). Cells were harvested at 24 h post-transfection and the endogenous miR-200c level was measured using RT-PCR and normalized to U6 mRNA. **B**. The data from the MTT assay that was performed each day for 6 days after transfection with the miR-200c mimic and the inhibitor. Each value represents the Mean ± SEM for at least three observations. *P<0.05 compared with controls. **C**. Flow cytometric analyses of the cell cycle of each treatment group. **D**. Flow cytometric analysis showing percent apoptosis in each treatment group. **E**. The number of migrated cells in each group in the *in vitro* transwell invasion assay is compared to the appropriate controls. Data are Mean ± SEM; n=3; *P=0.0015. **F**. The data from the wound-healing assay are shown. The upper panel shows the miR-200c INC or inhibitor group at 0h. The bottom panel shows the miR-200c INC or inhibitor group at 24h. The changes at the wound edges are illustrated with broken lines. Data are Mean ± SEM; n=3; *P=0.008.

To study the cellular function of miR-200c in NPC, we transfected the miR-200c mimic (200cM) and the miR-200c inhibitor (200cI) with their appropriate negative controls into 5-8F NPC cells. As expected, we found that the mimic increased the levels of miR-200c whereas the inhibitor decreased its levels (Figure 3A). To study the effect of miR-200c on growth of 5-8F cells, we performed the MTT assay. We found that the miR-200c inhibitor significantly decreased the viability of the 5-8F cells between days 3 and 6 post treatment, whereas the miR-200c mimic significantly increased the viability of the 5-8F cells suggesting that the miR-200c levels regulated NPC cell viability.

Since the 5-8F cell line is highly metastatic,we studied the effect of miR-200c inhibitor on the cell cycle and cell invasiveness. Cell cycle analysis showed that inhibition of miR-200c by the 200cI arrested the cells in G0-G1 phase. We found increased cells in the G0–G1 phase (42% to 66%), reduced number of cells in the S phase (30% to 17%) and no effect on the G2–M phase of the cell cycle (Figure 3C). In addition, we observed enhanced apoptosis of 5-8F cells (Figure 3D). These data therefore suggested that miR200c is required for cell cycle progression and survival.

To study the effect of miR-200c on the migration and mobility of the 5-8F cells, we performed an *in vitro* cell invasion assay based on the Boyden chamber assay. We observed that when treated with the miR200c inhibitor, 200cI, the number of 5-8F cells migrating through the matrigel decreased significantly in comparison to the control group (P<0.05; Figure 3E). Also, when we performed the *in vitro* scratch wound healing assay to study the effect of miR-200c on cell migration, we found that the 5-8F NPC cells migrated significantly slower in the miR-200c inhibitor group (P<0.05; Figure 3F). These results therefore suggested that miR-200c regulated the cellular migration and invasion properties of the NPC cells.

**Figure 4:**
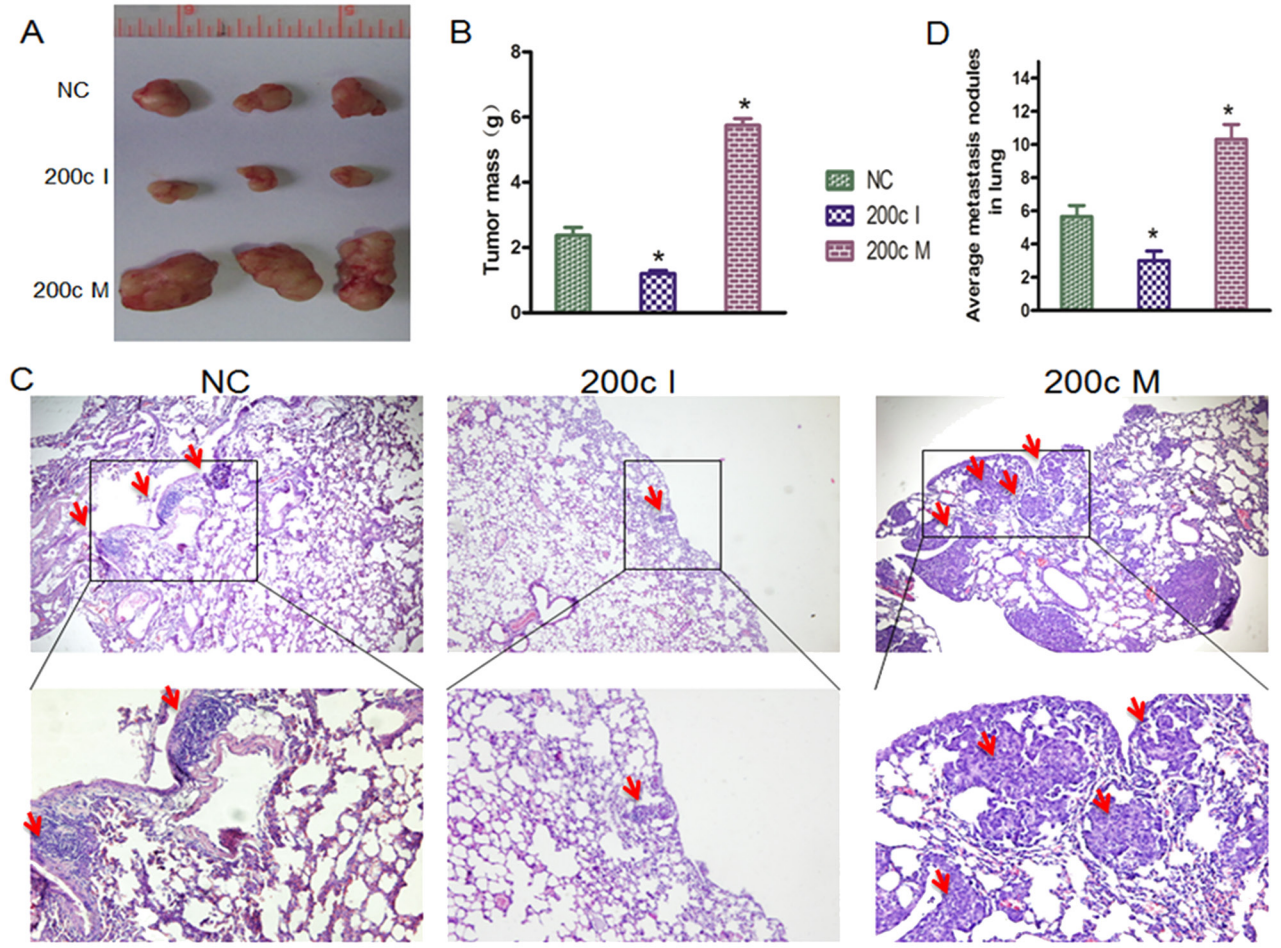
MiRNA-200c accelerates tumour growth and increases the distal pulmonary metastases *in vivo* **A**. Photographs of mice injected with 200cM/5-8F, 200cI/5-8F and NC/5-8F. **B**. Tumour weight averages between 200c M/5-8F, 200c I/5-8F and NC/5-8F mice groups at the endpoint of the experiment (day 35). Data are presented as Mean ± S.D. (n=3). NC/5-8Fvs 200c I/5-8F (P=0.011), NC/5-8F vs 200c M/5-8F (P=0.005). **C**. HE staining of lung tissue isolated from nude mice that had been injected with 200cM/5-8F, 200cI/5-8F or NC/5-8F via lateral tail veins (×200 magnification). The metastasis nodules are indicated by arrows. **D**. The graph shows the average of metastatic nodules in the lung of mice that received lateral tail injections of each cell line. Each group had three mice. Fisher's exact test: NC/5-8F vs 200c I/5-8F (P = 0.039), NC/5-8F vs 200c M/5-8F (P = 0.0135).

We then performed tumor xenograft and pulmonary metastasis studies with 5-8F NPC cells in BALB/c nude mice to investigate the role of miR-200c *in vivo*. Towards this, the 5-8F cells were collected after transfection with miR-200c mimic (200cM), miR-200c inhibitor (200cI) and negative control (NC)for 24h. For tumor xenograft studies, 200cM/5-8F, 200cI/5-8F and NC/5-8F were injected subcutaneously into the axillary fossae of the male BALB/c nude mice. When the tumors were analyzed 5 weeks post xenografting, the tumor volume from 200cI/5-8F cells was smaller than those from the NC/5-8F cells (Figure 4A). On the other hand, we found that the tumor volume from 200cM/5-8F cells was larger than those from NC/5-8F cells (Figure 4B). For pulmonary metastasis assays, 200cM/5-8F, 200cI/5-8F and NC/5-8F were injected into nude mice through the lateral tail vein. After 5 weeks, we found that the number of mice with lung metastases were lower in the group injected with 200cI/5-8F cells compared with the group injected with the NC/5-8F cells, whereas, the number of mice with lung metastases were higher in the group injected with 200cM/5-8F cells when compared to the group injected with the NC/5-8F cells (Figures 4C-4D). Together, these data suggest that miR200c accelerates the growth of 5-8F engrafted tumors and increases the distal pulmonary metastases *in vivo*.

### MiR-200c suppresses PTEN expression by targeting its 3′UTR

**Figure 5:**
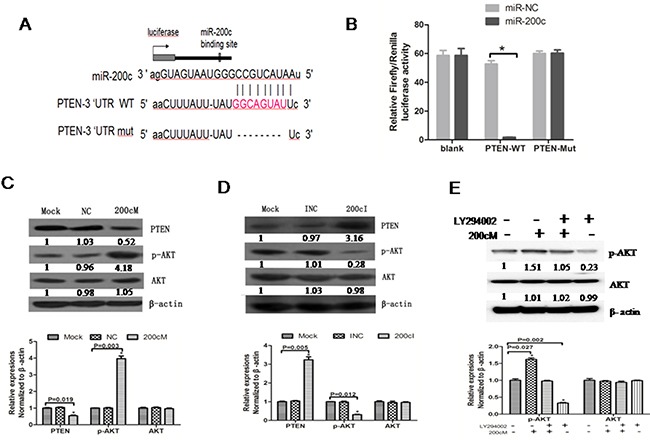
MiR-200c regulates PTEN-AKT signalling pathway in 5-8F NPC cells **A**. Schematic representation of the three reporter constructs named blank, 200c /PTEN WT and 200c/PTEN Mut. ‘Mut’ indicates the mutant construct in which the predicted binding site of miR-200c was deleted. **B**. The firefly luciferase vector was co-transfected with the indicated material (no treatment, 10 pmol NC, 10 pmol 200cM) into the HEK293 cells. Luciferase activities were measured after 48 h, and β-galactosidase was used to normalise for differences in transfection efficiency. Data was confirmed in duplicate experiments. The data are presented as the Mean ± SEM of separate transfections (n=6); *P = 0.021 compared with no treatment group. **C**. PTEN protein expression levels were increased and p-AKT protein levels were decreased upon transfection with 200cI compared to control group in 5-8F cells. **D**. PTEN protein expression levels were decreased and p-AKT protein levels were increased upon transfection with 200cM compared with the control group in 5-8F cells. **E**. The p-AKT protein level upon transfection with 200cM was similar to that obtained upon treating the cells with the AKT inhibitor (LY294002).

Since miR-200c promoted tumorigenesis in NPC, we wanted to identify the tumor suppressor genes that were inhibited by miR-200c. We found PTEN as one of the miR-200c-targeted genes in the TSGD (Tumor Suppressor Gene Database). To understand the interaction between PTEN and miR-200c, we cloned the 3′UTR of *PTEN* into a vector downstream of a firefly luciferase reporter gene with (PTEN WT) or without (PTEN Mut) the predicted miR-200c binding site (Figure 5A). Further, we transfected the HEK-293 cells with either of the constructs along with 200cM or NC. We found that the 3′UTRs of PTEN WT significantly decreased luciferase activities (P<0.05) in the 200cM transfected cells, whereas the PTEN Mut showed diminished regulation of miR-200c. These results showed that miR-200c inhibited PTEN by directly interacting with 3′UTR of PTEN (Figure 5B).

**Figure 6:**
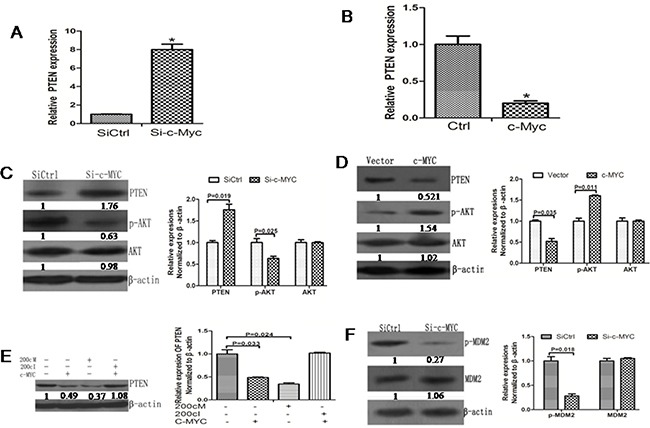
c-Myc promotes AKT signaling pathway activation by inhibiting PTEN expression **A**. RT-PCR data showing increased PTEN mRNA levels upon knockdown of c-Myc. **B**. RT-PCR data showing decreased PTEN mRNA levels upon c-Myc overexpression. **C**. Western blot data showing increased PTEN protein levels upon c-Myc knockdown. The fold-changes in the PTEN protein levels were calculated relative to the control. Data is presented as the Mean ± SD (N=3, *p<0.05). **D**. Western blot data showing decreased PTEN protein level upon c-Myc overexpression. The fold-changes in the PTEN protein levels were calculated relative to the control. Data is presented as the Mean ± SD (N=3, *p<0.05). **E**. The PTEN protein expression remained relatively similar upon co-expression of 200cI with c-MYC. The fold-changes in the PTEN protein levels were calculated relative to the control. Data is presented as the Mean ± SD (N=3, *p<0.05). **F**. p-MDM2 levels were lower due to knockdown c-Myc in the 5-8F cells. The fold-changes in the p-MDM2 protein level were calculated relative to the control. Data is presented as the Mean ± SD (N=3, *p<0.05).

PTEN is a vital tumor suppressor gene that functions by inhibiting the PI3K pathway and that regulates multiple biological processes like apoptosis, metabolism, cell proliferation and cell growth [25, 26]. Since miRNAs down regulate their targets by affecting mRNA translation or mRNA stability, we wanted to detect whether transfection with 200cI or 200cM affected the PTEN and p-AKT protein levels. Our analyses revealed that PTEN was upregulated and p-AKT was downregulated by the miR200c inhibitor, 200cI (Figure 6C), whereas PTEN was downregulated and p-AKT was upregulated by the miR200c mimic, 200cM (Figure 5D). The p-AKT protein level in 200cM treated cells was comparable to cells treated with the AKT inhibitor LY294002(40μmol/L) (Figure 5E). These results demonstrated that miR-200c upregulated the AKT signaling pathway by inhibiting PTEN.

### c-Myc activates the AKT pathway by repressing PTEN

Since we identified *PTEN* as the target gene of miR-200c and since miR-200c is regulated by c-Myc, we wanted to study if c-Myc could affect the PTEN and p-AKT expression levels similar to miR-200c. We observed that the PTEN mRNA level was upregulated upon knockdown of c-Myc upon analysis by RT PCR (Figure 6A). Conversely, the PTEN mRNA was downregulated upon overexpression of c-Myc (Figure 6B). The Western blot data were consistent with the RT-PCR results (Figure 6C–6D). Co-expression of 200cI with c-Myc abolished the increase in PTEN expression induced by c-Myc alone (Figure 6E). Furthermore, the phosphorylation of the AKT target MDM2 was also inhibited by the knockdown of c-Myc (Figure 6F). These data showed that c-Myc modulates the AKT signaling pathway via miR200c regulation of PTEN.

## DISCUSSION

**Figure 7:**
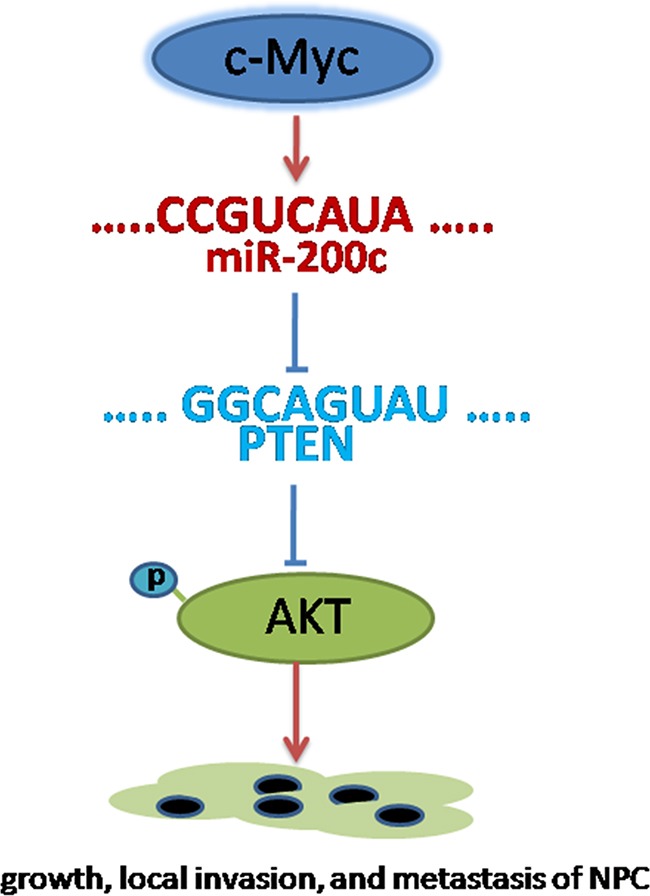
Conceptual relationships between miR-200c and nasopharyngeal carcinoma miR-200c can be activated by c-myc. Once activated, miR-200c can promote cell migration and invasion through targeting the PTEN 3′UTR and subsequently activating the AKT signaling pathway.

The critical role for c-Myc activity in cancer development and progression is now well established [2]. It is clear that a variety of non-coding miRNAs are regulated by c-Myc and in turn target a number of key cellular regulatory genes [5, 25, 26]. Results of this study revealed a crucial role of miR-200c as a novel target of c-Myc in c-Myc mediated cellular transformation. The oncogenic function of miR-200c is attributable at least in part to the inhibition of PTEN, a tumour suppressor candidate, through targeting the PTEN 3′UTR and subsequently activating the AKT signalling pathway (Figure 7).

The role of miR-200c in NPC has not been well studied although it has been found to play a role in many cancers [27–30]. We found that miR-200c was upregulated in NPC tissue samples in comparison with NPE samples. Also, since the siRNA downregulation of c-Myc decreased miR-200c expression we postulated that miR-200c expression is induced by c-Myc during different stages of tumorigenesis. Further, we found that inhibition of miR-200c reduced cell viability, cell cycle arrest in the G0–G1 phase and decreased cell migration and invasiveness. These results suggest that miR-200c is a c-Myc target that plays a significant role in NPC tumour genesis. In addition to our findings, miR-200c has been shown to be regulated by the oncogenic KRAS and PPARα [31].

To understand the role of miR-200c in initiating the EMT program, we screened for potential targets and found that the tumor suppressor gene, *PTEN*, was a direct target of miR-200c. *PTEN* is one of the most common tumor suppressor genes found altered either by mutation or deletion in human cancers including glioblastoma, endometrial cancer, and prostate cancer [32, 33]. PTEN is a protein tyrosine phosphatase that negatively regulates intracellular levels of phosphatidylinositol-3, 4, 5-trisphosphate in cells by dephosphorylating it and hence negatively regulates the AKT/PKB signalling pathway [34]. Phosphorylation of AKT is necessary for the activation of a cascade of multiple protein targets that are involved in cell growth, proliferation and invasion, and hence promote tumorigenesis. Our study demonstrates that miR-200c upregulation can result in higher AKT phosphorylation through its inhibition of PTEN expression. Apart from our finding, recently the miR-18 cluster has been reported to PTEN [35]. Since PTEN negatively regulates AKT activity, our data suggests a synergistic mechanism involving miR200c in creating the metastatic state [36]. The novel mechanism we propose is that c-Myc activation induces miR-200c that negatively regulates the expression of tumor suppressor genes like PTEN resulting in accelerated cellular growth, invasiveness, and metastasis of NPC. Although downregulation of PTEN and subsequent activation of AKT signalling is necessary for miR-200c to facilitate metastasis, there may be exist other downstream targets of miR-200c required for metastasis progression that need to be identified and investigated by future studies.

In summary, we have identified miR-200c as a pro-metastatic miRNA and a negative regulator of the key metastasis suppressor, PTEN. Our results revealed that the miR-200c expression was upregulated by c-Myc in NPC cells, and the upregulation of miR-200c in turn suppressed the expression of PTEN, leading to the activation of AKT signalling pathway, which ultimately promotes carcinoma cell motility and invasiveness. Although PTEN downregulation and AKT signalling activation appear to be necessary for miR-200c to facilitate metastasis, it is possible that additional miR-200c targets are involved in the miR-200c promoted metastatic progression. In this regard, further investigation on the functional characterization of miR-200c in tumour cells is warranted.

## MATERIALS AND METHODS

### Cell culture and stable transfection

The Cancer Centre of Sun Yet-Sen University (Guangzhou, China) kindly provided the NPC cell line 5-8F for the present study. We generated the 5-8F/Si-c-Myc cells, by siRNA knockdown of c-Myc and the negative control (NC) cells (5-8F/Si-control) with a negative mock siRNA vector using the strategy as previously reported [19]. All the 5-8F cell lines were cultured in RPMI-1640 medium supplemented with 10% fetal bovine serum, and maintained in a humidified atmosphere of 5% CO_2_ in air at 37°C.

### Patient samples and LCM

For the miRNA expression study, 10 normal nasopharyngeal epithelium (NPE) samples and 10 nasopharyngeal carcinoma (NPC) biopsies were obtained from Xiangya Hospital of Central South University (Changsha, China). Normal nasopharyngeal epithelium samples were obtained from biopsy-negative cases. The clinical and histopathological analysis of the NPC samples was performed at the Xiangya Hospital of Central South University (Changsha, China). The tissue samples were snap frozen in liquid nitrogen and stored at -80°C until being subjected to laser capture micro-dissection (LCM). The samples were fully encoded and examined according to the protocol approved by the Institutional Review Board of Human Subjects Research Ethics Committee. All the individuals participating in this study were provided with informed consent.

8μm-thick frozen sections of fresh NPC and NPE were prepared using a Leica CM 1900 cryostat (Lecia, Germany) at -25°C and LCM was performed as previously described [20].

### The miRNA microarray analysis

To identify differences in miRNA profile between the 5-8F/Si-control and the 5-8F/Si-c-Myc cell lines, the miRNA microarray analysis was performed using a miRNA microarray obtained from from Capital Bio Corporation (Beijing, China) and composed of 434 human (containing 122 predicted miRNA sequences from a published reference), 196 rat and 261 mouse miRNAs that were registered in the Sanger miRBase miRNA database (http://www.mirbase.org/; miRBase Release 8.2). The miRNA microarray used was a single-channel fluorescence chip with all oligonucleotide probes being labelled with Cy3 fluorescent dyes (green colour). The miRNAs were enriched from total RNA-extracted cells (5-8F/Si-c-Myc and 5-8F/Si-control) using a mirVana miRNA Isolation Kit (Ambion, Foster City, CA) and labelled using a mirVana Array Labelling Kit. Labelled miRNAs were then hybridized to miRNA microarrays that had 509 probes in triplicate to determine differential expression between the cell lines. This procedure was repeated twice. Fluorescence scanning was performed using a double-channel laser scanner (LuxScan 10K/A; CapitalBio). Figure signals were transformed to digital signals using image analysis software (LuxScan3.0; CapitalBio). Raw data were normalized and analyzed using the Significance Analysis of Microarrays software (SAM, version 2.1; Stanford University, CA).

### siRNAs transient transfection

MiR-200c mimics (200c M) and miR-200c inhibitors (200c I) in the form of a short duplex siRNA duplex were synthesized and purified by Shanghai Gene-Pharma Co. (Shanghai, China). siRNA duplexes with non-specific sequences were used as the negative control (NC) and as the inhibitor negative control (INC), respectively. The four different siRNAs (200cM, 200cI, NC and INC) were transfected into separate group of 5-8Fcells using Lipofectamine 2000 (Invitrogen) in accordance with the manufacturer's protocol.

### RNA isolation and quantitative RT-PCR analysis

Total RNA was isolated using TRIZOL reagent (Invitrogen, USA) according to the manufacturer's instructions. RNA was extracted with the RNeasy kit (Qiagen, Valencia, CA) and treated with DNase I according to the manufacturer's instructions (Qiagen, Valencia, CA). The integrity and quality of RNA was confirmed by agarose gel electrophoresis and absorbance at 260 nm. Total RNA was then reverse transcribed to cDNA using the SuperScript™ First-Strand Synthesis System with random hexamer primers ( Promega, USA ).

Real time RT-PCR was performed to detect cellular miRNAs as previously described [21]. The primers for RT-PCR to detect miRNA were designed based on the miRNA sequences provided by the Sanger Center miRNA Registry and were synthesized and purified by the Shanghai Gene-Pharma Co. (Shanghai, China). Real-time PCR was performed on the BIO-RAD IQTM5 Multicolor Real-time PCR Detection System (Bio-Rad). U6 RNA was used as an endogenous control for miRNA detection. All reactions were run in triplicate.

### Northernblot analysis

Northern blot analysis was performed to detect cellular miRNAs [22]. The blot was reprobed for U6 to control for equal loading and quantitation performed with a Storm 860 phospho imager (Molecular Dynamics) and Image Quant software.

### Western blot analysis

Western blotting was performed as described previously [23]. Anti-β-actin was purchased from Sigma-Aldrich (St Louis, MO. 1:1000 dilution). The anti-PTEN, anti-MDM2, and anti-phospho-MDM2 were from Santa Cruz Biotechnology (Santa Cruz, CA. 1:400 dilution). The anti-c-Myc was from Upstate Biotechnology (Chicago, IL. 1:1000 dilution). The anti-AKT and anti-phospho-AKT were from Cell Signaling Technology (Danvers, MA. 1:1000 dilution). The loading control for Western blotting was β-actin.

### 3′UTR luciferase reporter assays

The binding site of the miRNAs and 3′UTR of the target genes were as predicted by TargetScan 5.0 and Pictar. We synthesized single strands of 3′UTR of the target genes that contained the binding site of the miRNAs. Single strands of the 3′UTR of the target genes that deleted five bases in the binding site of the miRNAs were then synthesized as a mutant control. The oligonucleotides used in these studies were as follows: 3′UTR of mutant PTEN [which deleted eight bases (GGCAGTAT) in the binding site of miR-200c]: AATTAAAACTTTATTTATGGCAGTATTCATAATTAGCCTGAAATGCAU. The oligonucleotides were digested with HindIII and SpeI and ligated into pmiR-Report luciferase vector (Ambion, Austin, TX). Three 3′UTR luciferase reporters were constructed and named blank, PTEN WT, and PTENMut (Figure 3B).

Further, the 5-8F cells were seeded in 24-well plates 24 h prior to transfection. The following day, 200ng of reporter plasmid and 10 pmol of miRNA mimic or mimic-NC were co-transfected into cells using Lipofectamine 2000. Luciferase activity was measured in cell lysates 24 h after transfection using a Luciferase Assay kit (Promega, Madison, WI). β-Galactosidase activity was measured in cell lysates using the β-galactosidase Enzyme Assay System (Promega). The results were normalized against β-galactosidase activity.

### 3-(4,5-Dimethylthiazole-2-yl)-2,5-biphenyl tetrazolium bromide assay

After the transient transfection with miRNA mimics or inhibitors, the cells (4×10^3^) were seeded into 96-well culture plates. Cell viability was determined daily for 6 days using the (3-(4, 5-Dimethylthiazole-2-yl) -2, 5-biphenyl tetrazolium bromide; Sigma-Aldrich) assays were performed daily for 6 days. In brief, 0.025 ml of MTT solution (5 mg/ml) was added to each well, and the cells were incubated for 4 h. After centrifugation, the supernatant was carefully removed from each well. The coloredformazan crystals produced from the MTT were dissolved in 0.15 ml of dimethyl sulfoxide and mixed for 10 min. The optical density (OD) value was measured at 570 nm with an enzyme-linked immunosorbent assay reader.

### Flow cytometric analysis of the cell cycle

The cells were collected and washed twice with phosphate-buffered saline followed by fixation in 70% ethanol overnight. The cells were then centrifuged at 1500 g for 8 min, resuspended in 50μg/ml propidium iodide (Sigma-Aldrich) in phosphate-buffered saline and immediately analyzed in the FACStar flow cytometer (Beckton-Dickinson, Mountain View, CA). Appropriate settings of forward and side scatter gates were used to examine 10 000 cells per experiment. Data were analysed using ModFit software (Verity Software House, Topsham, ME). Values were expressed as the mean and error deviation of three independent experiments.

### Flow cytometric analysis of apoptosis

Cells were harvested, washed twice with PBS and double stained with FITC-conjugated Annexin V and PI. The cells were analysed by quadrant statistical analysis. The Annexin V FITC was highlighted in green, and PI was highlighted in red.

### Wound-healing assay

To demonstrate the effect of miR-200c on the migration and mobility of the 5-8F cells, Wound healing assay was performed to evaluate 5-8F cell motility in response to the depletion of miR 200c. At 48 h after transfection with miRNA200c inhibitors, a total of 5×10^5^ 5-8F cells were plated into a6-wellplate and left overnight to achieve sub-confluence. After scraping the cells with a 10μl pipette tip vertically to the marked lines and washing with PBS three times to get rid of floating cells, a cell-free space was created. Images were taken at 0 h and 24 h after wounding under the inverted microscope.

### Transwell invasion assay

To demonstrate the effect of miR-200c on the migration and mobility of the 5-8F cells, an in vitro cell invasion assay was performed based on the principle of the Boyden chamber assay. In brief, cell invasion was measured by a Matrigel invasion chamber assay. At 48 h after transfection with miRNA mimics or inhibitors, 5-8F cells (1× 10^4^cells per well) were seeded onto the upper compartment of the transwell insert membrane of a 24-well culture plate, which was coated with a uniform layer of dried basement membrane matrix solution (EC matrix, Chemicon, Temecula, CA). Fetal bovine serum was added to the lower chamber as a chemoattractant. Following another 48 h of incubation, cells that remained on the top of the membrane were scrubbed off, while the migrated cells on the lower surface of membrane were fixed in methanol, stained with crystal violet and air dried. Numbers of migrated cells on the entire membrane were counted manually under the inverted microscope.

### *In vivo* tumorigenesis

A subcutaneous tumor xenograft model and a tumor mestastasis model were used to further investigate the function of miR-200c *in vivo*. All animal procedures were performed in accordance with institutional guidelines. In brief, five-week-old male BALB/c nude mice were randomly divided into three groups: (1) miR-200cmimics (200c M), (2) miR-200c inhibitors (200c I) and (3) negative control (NC). To examine the effect of miR-200 on tumorigenesis, the subcutaneous xenograft model was established by injecting 1× 10^6^ 5-8F cells transfected with either 200c M, 200c I or NC that were suspended in 200 μl phosphate-buffered saline into the axillary fossae of individual animals (n = 3 per group). On Day 35 after injection, mice were euthanized, and tumors were excised and weighted. To examine the ability of miR-200c to enhance pulmonary metastasis, 1× 10^6^ 5-8F cells transfected with 200c M, 200c I or NC were injected into the lateral tail veins of individual nude mice under isoflurane anesthesia (n = 3 per group). Five weeks after injection, the animals were euthanized and lungs were perfused and fixed with phosphate-buffered neutral formalin before paraffin embedding; and 5-μm sections were stained with hematoxylin and eosin. The metastases were counted in a double-blind manner with the aid of a dissecting microscope (Nikon, Tokyo, Japan).

### Data analysis

Statistical analyses were carried out using the SPSS version 15.0. The results are expressed as the mean± S.E.M. Comparison of means between two groups was performed using the two-tailed unpaired Student's t-tests. A two sided P-value of less than 0.05 was considered statistically significant.
